# Multi-Functional Boron-Delivery Agents for Boron Neutron Capture Therapy of Cancers

**DOI:** 10.3390/cancers15133277

**Published:** 2023-06-21

**Authors:** Sebastian O. Oloo, Kevin M. Smith, Maria da Graça H. Vicente

**Affiliations:** Department of Chemistry, Louisiana State University, Baton Rouge, LA 70803, USA; soloo1@lsu.edu (S.O.O.); kmsmith@lsu.edu (K.M.S.)

**Keywords:** boron, BNCT, anticancer agents

## Abstract

**Simple Summary:**

Many boron-containing compounds have been synthesized and proposed as boron-delivery agents for boron neutron capture therapy for cancers, including brain tumors and melanomas. However, only a few have been investigated in clinical studies, leaving their potential efficacy for cancer treatment unknown. With the recent availability of accelerator-based neutron sources, clinical trials are underway using the boronated amino acid (L)-4-hydroxyboryl phenylalanine. Simultaneously, there is renewed interest in the development of multi-functional boron-delivery agents for cancer diagnosis and treatment. Promising agents can deliver therapeutic amounts of boron into tumor cells with high specificity and low toxicity. Among the different classes of boron-containing compounds, peptide-, porphyrin-, liposome-, and nanoparticle-based-delivery agents are the most promising due to their tumor-targeting and concurrent imaging abilities. A systematic and comparative evaluation of these agents in clinical trials would allow the determination of their full potential for cancer treatment.

**Abstract:**

Boron neutron capture therapy (BNCT) is a binary cancer treatment that involves the irradiation of ^10^B-containing tumors with low-energy neutrons (thermal or epithermal). The alpha particles and recoiling Li nuclei that are produced in the ^10^B-capture nuclear reaction are high-linear-energy transfer particles that destroy boron-loaded tumor cells; therefore, BNCT has the potential to be a localized therapeutic modality. Two boron-delivery agents have been used in clinical trials of BNCT in patients with malignant brain tumors, cutaneous melanoma, or recurrent tumors of the head and neck region, demonstrating the potential of BNCT in the treatment of difficult cancers. A variety of potentially highly effective boron-delivery agents have been synthesized in the past four decades and tested in cells and animal models. These include boron-containing nucleosides, peptides, proteins, polyamines, porphyrins, liposomes, monoclonal antibodies, and nanoparticles of various types. The most promising agents are multi-functional boronated molecules and nanoparticles functionalized with tumor cell-targeting moieties that increase their tumor selectivity and contain a radiolabel or fluorophore to allow quantification of ^10^B-biodistribution and treatment planning. This review discusses multi-functional boron agents reported in the last decade, but their full potential can only be ascertained after their evaluation in BNCT clinical trials.

## 1. Introduction

Boron neutron capture therapy (BNCT) is based on the very high cross section of ^10^B nuclei for neutron capture (3838 barns), compared with those of biologically abundant nuclei such as ^12^C (0.0034 barn), ^1^H (0.33 barn), and ^14^N (1.8 barns). The ^10^B (n,α)^7^Li nuclear capture reaction produces excited ^11^B nuclei that undergo spontaneous fission to produce high-linear-energy transfer (high-LET), alpha particles (^4^He) and recoiling ^7^Li nuclei, along with 2.4 MeV of kinetic energy [1,2,3]. Since the high-LET particles have limited path lengths in tissue (5–9 μm), BNCT could be a localized therapeutic modality, capable of destroying ^10^B-containing tumor cells in the presence of ^10^B-free healthy cells.

BNCT has been used clinically for over 60 years for the treatment of difficult-to-treat cancers, including high-grade gliomas, recurrent head and neck tumors, and metastatic melanomas [4,5,6,7]. Due to the potential of BNCT, a large number of boron-containing drugs have been synthesized and investigated as BNCT agents, but just a handful have been tested in experimental tumor-bearing animals [8,9,10,11,12,13]. Of these, only a few have been investigated in BNCT clinical trials, due in part to the lack of availability and high cost of neutron sources. Until 2014, only specially designed nuclear reactors could produce thermal (<0.5 eV) or epithermal (0.5 eV to 10 keV) neutrons for BNCT trials. However, since then, accelerator-based neutron sources have been developed that could be installed in laboratories and hospitals, facilitating pre-clinical evaluation of potential boron-delivery agents, as well as patient treatment [6].

In general, a promising boron agent should deliver a therapeutic amount of ^10^B of about 30 µg ^10^B/g to target tumors, with high tumor-to-normal tissue (T/N) and tumor-to-blood (T/B) concentration ratios (>3:1), and low systemic toxicity [8,9,10,11,12,13]. In addition, the boron agent should persist within the tumor during the neutron irradiation treatment but be rapidly eliminated after treatment. To facilitate quantification of ^10^B distribution and treatment planning, the boron drug can be radiolabeled, for example, with ^18^F, or conjugated to a fluorophore without altering its pharmacokinetic properties. On the other hand, to increase tumor specificity and high T/N and T/B, the boron agent can be attached to a tumor-targeting moiety such as a peptide or monoclonal antibody. Boronated agents that accumulate in tumors based only on the enhanced permeability and retention (EPR) effect tend to display high boron concentration in the blood, which might cause side effects due to blood vessel damage upon neutron irradiation. For the treatment of brain tumors, it is also desirable that the boron agent be able to permeate across the blood–brain barrier (BBB) or be used in combination with BBB disruption drugs, such as mannitol or Cereport. Furthermore, the investigation of potential drug combinations, drug doses, delivery formulations, and administration procedures in clinical trials should be thoroughly investigated to ensure the success of BNCT [6]. The development of multi-functional agents can also allow for dual therapies, such as the combination of chemotherapy with BNCT [14,15].

The major challenges to date in compound development for BNCT have been the requirements for selective tumor-targeting with minimal normal tissue uptake, and for the delivery of therapeutic boron concentrations to tumors. Several classes of boron-containing compounds have been proposed for BNCT, including amino acids, peptides, proteins, polyamines, nucleosides, carbohydrates, porphyrins, liposomes, monoclonal antibodies, polymers and nanoparticles (e.g., quantum dots, gold, silica) [8,9,10,11,12,13]. The boron sources in these compounds are often the neutral isomeric carboranes—*ortho*-, *meta*-, and *para*-C_2_B_10_H_12_, the negatively charged *closo*-B_12_H_12_^2−^, *closo*-CB_11_H_12_^−^, *nido*-C_2_B_9_H_12_^−^, and [3,3′-Co (1,2-C_2_B_9_H_11_)_2_]^−^, due to their high boron content and ease of attachment to both small and large molecules and nanoparticles. In this review, we will focus on promising multi-functional boron-delivery agents that have been proposed for BNCT, based on currently available experimental cell and animal studies, with an emphasis on those reported in the last decade. 

## 2. Clinically Approved Agents for BNCT

**Figure 1:**
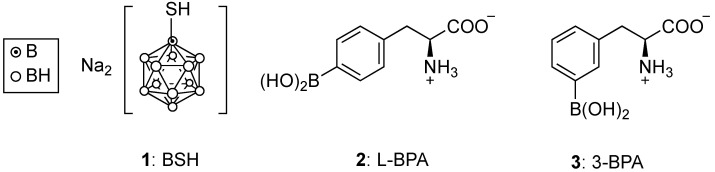
Structures of clinically approved agents for BNCT: BSH and L-BPA, and of recently reported 3-BPA.

Two boron-containing agents have been approved for clinical use, sodium mercaptoundecahydro-*closo*-dodecaborate (BSH, **1**) and (*L*)-4-dihydroxy-borylphenylalanine (L-BPA, **2**) [1,2,3,4,5,6], as shown in Figure 1. Despite the high boron content of BSH (60%), this agent lacks tumor specificity and was found to have lower BNCT therapeutic efficacy compared with L-BPA [6,11]. Therefore, L-BPA is the most used boron compound in BNCT clinical trials, usually delivered as a water-soluble fructose or sorbitol complex, ^10^B-enriched, and ^18^F-labeled to allow for positron emission tomography (PET) imaging prior to neutron irradiation. In 2020, L-BPA was marketed under the name Borofalan (Steboronine^®^) by the company Stella Pharma, as its d-sorbitol complex and 99% ^10^B-enriched. Despite its low boron content (5%), L-BPA is taken up preferentially in tumor cells by L-type amino acid transporter 1 (LAT1, SLC7A5), efficiently delivering boron to target tumors with T/N~2:1, following administration of up to 700 mg/kg dose in clinical trials.

Despite promising results for the clinical efficacy of L-BPA, several challenges remain, including its required high dosage for cancer treatment, low water solubility, and low tumor-targeting ability. Furthermore, solubilization of L-BPA with fructose or sorbitol cannot be used in patients with fructose intolerance. Therefore, the meta isomer of L-BPA, (*L*)-3-dihydroxy-borylphenylalanine or 3-BPA (**3**), was recently evaluated in various LAT1-expressing tumor cells (B16F10, T3M-4, U87MG, A549) and in BALB/c mice bearing B16F10 or T3M-4 subcutaneous tumors [16]. Comparative studies with L-BPA indicate that the 3-BPA isomer (**3**) has similar biodistribution and tumor-targeting ability, but superior water solubility, and therefore does not require a solubilizing sugar for administration.

Other boron-containing amino acids have been investigated as boron-delivery agents for BNCT. In particular, boron derivatives of naturally occurring L-tyrosine and L-dope amino acids, both precursors of melanin and substrates for tyrosinase, were synthesized and shown to have enhanced selectivity toward SK-23 melanoma cells with high tyrosinase expression [17]. Several boron derivatives of unnatural amino acids have also been investigated, revealing enhanced tumor uptake and persistence, but these tend to display increased toxicity and lower water-solubility compared with L-BPA [18]. 

## 3. Boronated Peptides and Monoclonal Antibodies

**Figure 2:**
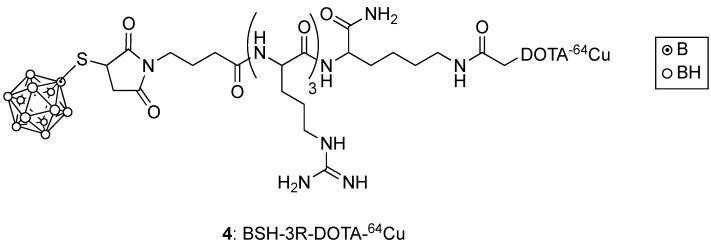
Structure of BSH-3R-DOTA-^64^Cu.

Boron-containing linear and cyclic peptides that target specific protein receptors overexpressed in cancer cells, and cell-penetrating peptides (CPPs) with multiple arginine residues, can potentially deliver the required high boron amount to tumor cells with higher T/N than L-BPA. Such targeting peptides have been directly functionalized with various boron clusters, and/or linked to other compounds, including porphyrins, liposomes, and nanoparticles to produce multi-functional agents. For example, BSH bearing three arginines and a ^64^Cu-DOTA ligand for PET imaging (shown in Figure 2), was shown to target U87 glioma tumors in mice more selectively compared with ^64^Cu-labeled BSH [19]. This BSH-3R-DOTA-^64^Cu (**4**) agent displayed a T/B of 2.8 at 6 h post-injection, and a similar tumor uptake to a BSH-11R derivative of larger molecular weight [20]. In a more recent study, an octa-arginine CPP bearing the D-enantiomer of arginine with enhanced stability and the mitochondria-targeting _D_[RLARLAR]_2_ peptide were conjugated to a carboxyl-functionalized *closo*-dodecaborane cluster [21]. The conjugate bearing the _D_[RLARLAR]_2_ peptide displayed mitochondria localization and four-fold increased uptake in C6 glioma cells, compared with BSH. On the other hand, the octa-arginine conjugate showed decreased cellular uptake and no mitochondria localization. Furthermore, thermal neutron irradiation of the C6 cells following treatment with the _D_[RLARLAR]_2_ conjugate revealed a significantly higher ATP depletion and cell-killing effect compared with the octa-arginine conjugate and with BSH. Therefore, although CPPs generally increase tumor cell uptake, their boronated derivatives display lower tumor-selectivity compared with receptor-specific targeting peptides [22,23]. 

**Figure 3:**
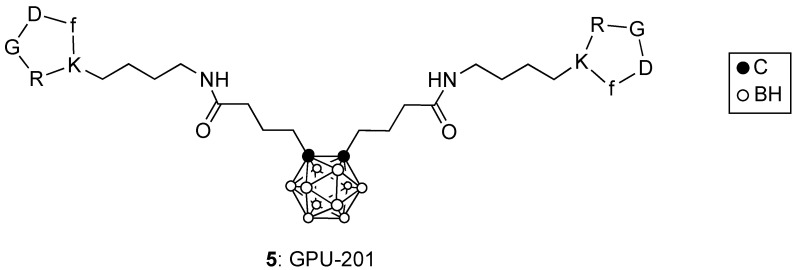
Structure of GPU-201.

RGD-containing peptides have been shown to bind to integrin αvβ3 overexpressed at the surface of tumor cells. The cyclic c(RGDfK) peptide attached to BSH or to *ortho*-carborane clusters were prepared and evaluated in mice bearing SCCVII tumors [24,25]. These conjugates showed high in vitro integrin αvβ3 affinity, and higher tumor uptake and retention compared with BSH. Furthermore, the *ortho*-carborane derivative conjugated to two c(RGDfK) peptides, designated GPU-201 (**5**) and shown in Figure 3, was administered IP to tumor-bearing mice and irradiated with a thermal neutron beam at the Kyoto University reactor, giving a significantly larger enhancement ratio in the Q cells and the total (Q + P) cell population compared with BSH [25]. The conjugation of c(RGDfK) to a cysteine residue of bovine serum albumin (BSA) functionalized with a maleimide-functionalized *closo*-dodecaborate (MID), produced the multi-functional cRGD-MID-BSA which was evaluated in U87MG (integrin αvβ3 positive) and A549 (integrin αvβ3 negative) cells [26]. Upon thermal neutron irradiation at the Kyoto University reactor, cRGD-MID-BSA produced higher cell-killing efficiency in the U87MG cells compared with L-BPA and MID-BSA, which showed similar effects. Furthermore, the Cy5-labeled conjugate, Cy5-cRGD-MID-BSA, was shown to selectively accumulate in U87MG gliomas and colon 26 tumors in mice, delivering a higher amount of boron to tumors than did Cy5-MID-BSA. Upon thermal neutron irradiation, the cRGD-MID-BSA conjugate showed enhanced tumor growth suppression than did MID-BSA [26].

A 36-amino acid neuropeptide Y has recently been functionalized with *meta*-carborane clusters, due to their higher stability compared with *ortho*-carborane, and evaluated in cells overexpressing hY_1_ receptors (MCF-7 and HEK293) [27]. The boron-rich neuropeptide was shown to have high selectivity and accumulation in hY_1_R-expressing cells and low cytotoxicity. The bombesin peptide sBB2L known to target the gastric-releasing peptide receptor (GRPR), has also been functionalized with *meta*-carboranes and sugar moieties, and tested in GRPR-expressing PC3 cells [28]. The conjugate was shown to be efficiently internalized into the PC3 cells and displayed low cytotoxicity. 

The specific targeting of EGFR-overexpressing cells has been achieved by using the monoclonal antibodies (mAbs) Cetuximab and L8A4 which target EGFR overexpressed in tumors such as gliomas, bearing up to 1000 boron atoms [29,30]. Boronated polyamidoamine dendrimers conjugated to mAb Cetuximab (BD-C225) or mAb L8A4 (BD-L8A4) were administered to rats bearing F98 gliomas; the highest BNCT efficacy was observed by using a combination of BD-C225 and BD-L8A4 administered by convection-enhanced delivery [29]. In addition, a study using rats with F98_npEGFR_ gliomas reported the best survival data using convection-enhanced delivery of BD-L8A4, either alone or in combination with L-BPA [30]. Although the use of mAbs has shown high tumor selectivity, some of the disadvantages compared to peptides are that antibodies are difficult to produce and derivatize, have poor tumor cell internalization, and are expensive to obtain. 

## 4. Porphyrin Derivatives for BNCT

Porphyrin-based compounds offer high promise as boron-delivery agents since some of these derivatives are already used in the clinic for the treatment of cancer and other diseases using photodynamic therapy (PDT). The intrinsic fluorescence properties of this type of compound allows for fluorescence-based detection and quantification of boron accumulated in blood, tumors, and other tissues. Furthermore, porphyrins form robust complexes with a variety of metal ions, including ^64^Cu(II), which allows their use in PET and other imaging technologies. Development of sensitizers for clinical application in PDT requires access to relatively large quantities of drug precursors. Thus, most successful porphyrinoid sensitizers have been synthesized from natural materials that are available in bulk (e.g., animal blood, leaves, algae, yeasts); alternatively, easily synthesized porphyrinoids such as 5,10,15,20-tetraphenylporphyrin (TPP) and phthalocyanine (Pc) derivatives have been used [31].

**Figure 4:**
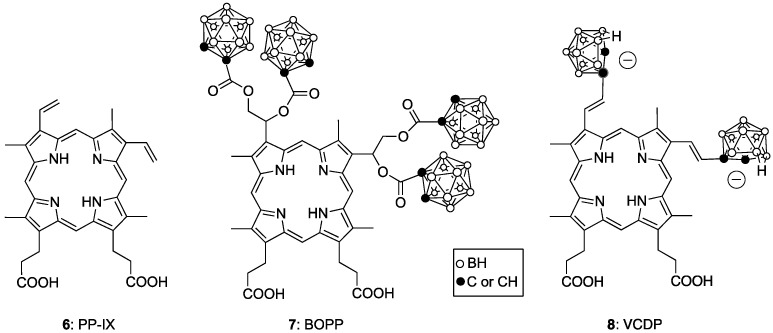
Boronated derivatives of protoporphyrin-IX **6**.

So-called purified hematoporphyrin derivative (Photofrin, porfimer sodium) was the first porphyrin derivative to be approved by the FDA for use in PDT. It is a complex mixture of substances derived eventually from hemin via protoporphyrin-IX (**6**, PP-IX). It is used for the treatment of lung, esophageal, bladder and cervical cancer, as well as for melanoma [32]. Several boronated derivatives of PP-IX have been proposed as boron agents for BNCT [33], for example, BOPP (**7**) [34] and VCDP (**8**) [35], as shown in Figure 4. These porphyrins were shown to have high tumor-cell uptake, due in part to their high affinity for the low-density lipoprotein (LDL) receptors, which are upregulated in many tumors, including gliomas. The tumor boron content was further enhanced by the co-administration of BOPP and L-BPA, and by delivery through convection-enhanced procedures.

**Figure 5:**
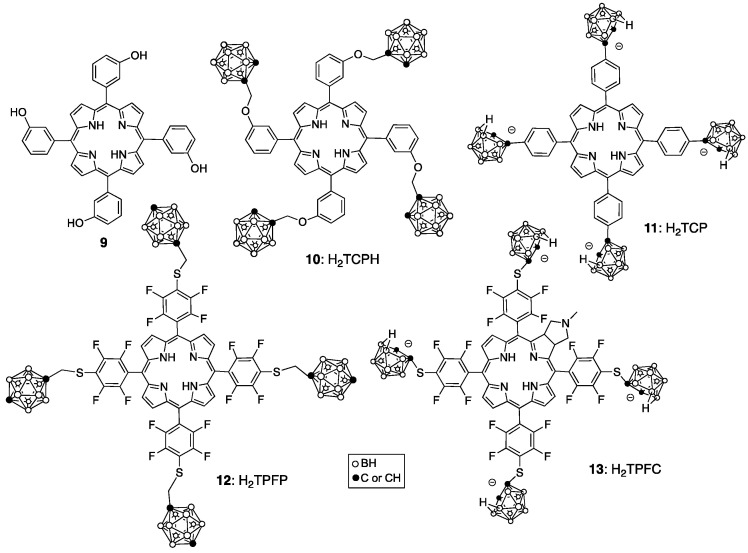
Boronated derivatives of 5,10,15,20-tetra(3-hydroxyphenyl)porphyrin **9**.

5,10,15,20-Tetra(3-hydroxyphenyl)chlorin (Foscan), synthesized from the corresponding 5,10,15,20-tetra(3-hydroxyphenyl)porphyrin (**9**), is currently approved in Europe for the treatment of head, neck, prostate, pancreatic and breast cancers [36]. It has the advantage that an intense long-wavelength absorption is present in the chlorin chromophore and this enables the use of long-wavelength light which travels deeper into tissues. It also is a mixture but has been fully characterized as four atropisomers (due to hindered rotation about the chlorin-hydroxyphenyl bonds). Boronated derivatives of **9** have been investigated as BNCT agents, including H_2_TCPH (**10**) [37], H_2_TCP (**11**) [38,39] and their metalated (copper and zinc) complexes (Figure 5). The Zn(II) metalloporphyrins often show enhanced fluorescence, while the Cu(II) complexes allow for tumor imaging via ^64^Cu-PET. The tetra-anionic H_2_TCP displays enhanced water solubility compared with the neutral porphyrin derivatives, low toxicity, and high tumor cell uptake. A porphyrin analog of H_2_TCP bearing eight boron clusters directly attached to the porphyrin ring, designated H_2_OCP, was also reported [40]. Although this derivative features a high boron content by weight and was shown to have low toxicity toward V79 lung and human glioma T98G cells, its tumor cell uptake was lower than that observed for H_2_TCP (**11**). Other porphyrin derivatives of high boron content, bearing up to 16 boron clusters via attachment of cobaltabis(dicarbollides) have been proposed as boron-delivery agents for BNCT [41]. The cellular uptake of these derivatives into HEp2 cells was shown to increase with the number of cobaltabis(dicarbollide) groups, up to six. Toxicity studies in Balb/c mice revealed maximum tolerated doses (MTD) above 150 mg/kg [42]. Enhanced tumor cell uptake of boron-containing porphyrins can be achieved by conjugating a cell-penetrating peptide to the porphyrin ring [43].

The fluorinated porphyrin H_2_TPFP (**12**) and several of its derivatives attached to a triethylene glycol or polyamine chain were investigated and shown to have low toxicities and high uptake into T98G glioma cells [44]. These fluorinated porphyrin analogs could be used for dual imaging by fluorescence and ^18^F-PET. The chlorin derivative H_2_TPFC (**13**) showed enhanced water solubility and a stronger long-wavelength absorption around 640 nm compared with 12. H_2_TPFC (**13**) was shown to deliver a higher amount of boron to melanotic melanoma B16F1 and rat glioma F98 cells, and to tumor-bearing mice, than L-BPA [45]. A significant inhibition of tumor growth was observed upon light irradiation 3 h after IV injection of H_2_TPFC (**13**) into mice, and administration via CED in combination with L-BPA enhanced the BNCT effect compared with H_2_TPFC or L-BPA alone [45].

Chlorin, bacteriochlorin, and phthalocyanine derivatives are promising as dual sensitizers for both BNCT and PDT because they display intense long-wavelength absorption in the red region of the visible spectrum where tissues have increased transparency to light. For example, mono-aspartylchlorin e_6_ (NPe6, LS-11, Talaporfin, Laserphyrin) has been used as a PDT photosensitizer in multiple clinical trials [46]. The current source of the chlorophyll a that is used for synthesis of NPE6 is the *Spirulina* alga which does not produce any chlorophyll b (unlike spinach, the earlier source, which produces both chlorophyll a and b). This sensitizer is used for colorectal cancer, and glioma. Another chlorophyll derivative, HPPH (Photochlor), enjoys the long-wavelength absorption maximum (665 nm) characteristic of the chlorin chromophore and has clinical applications in PDT treatment of basal cell carcinoma, lung, esophageal and head/neck cancers [47]. The bacteriochlorin chromophore absorbs at an even longer wavelength than chlorophyll derivatives; for example, the palladium complex TOOKAD is useful for PDT treatment of prostate cancer, and absorbs at 763 nm [48].

**Figure 6:**
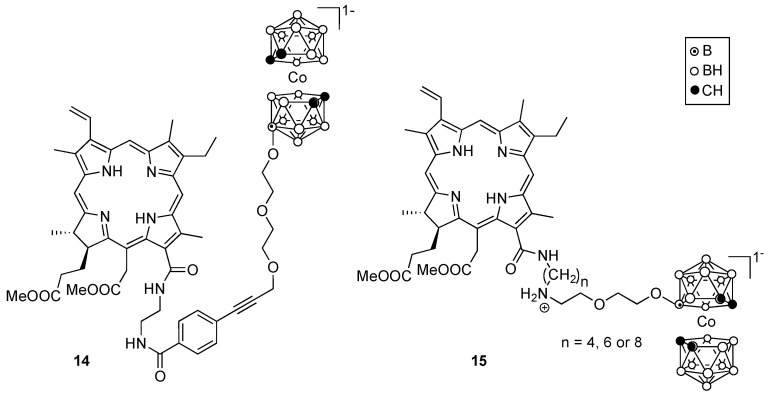
Representative boronated derivatives of chlorophyll a.

Several boron-containing chlorophyll a derivatives have been investigated [49], including chlorins **14** and 1**5**, shown in Figure 6. Similar to porphyrins, this type of compound also binds to LDL [50], and as a result typically shows preferential accumulation in tumor cells. These compounds exhibited high phototoxicity in human lung A549 cells upon light irradiation [51]. Studies in tumor-bearing mice showed that compound **15** accumulated preferentially in the tumor with a T/N ratio of about 3:1, 3 h after IV injection of the sensitizers [52].

**Figure 7:**
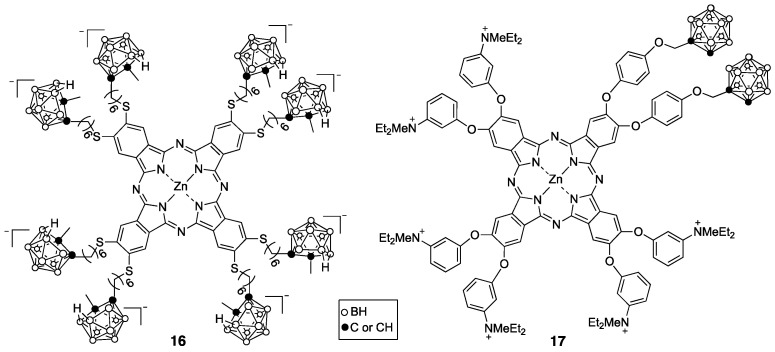
Representative boronated derivatives of Pcs.

Phthalocyanines (Pcs) are readily synthesized, feature high chemical stability, and have many commercial applications, including use as non-bleaching automobile paint. The aluminum complex of phthalocyanine tetrasulfonate (Photosens) absorbs above 675 nm and has been approved in Russia for PDT treatment of skin, mouth, breast, stomach and intestinal cancers [53]. Though relatively easy to synthesize, this compound is a mixture related to various possible positions for the sulfonic acid groups on the fused benzene rings and tends to persist in the skin for weeks after administration. Several boron-containing Pcs [54,55] and related porphyrazines [56] have been reported as potential PDT and BNCT sensitizers, including anionic and cationic derivatives such as **16** and **17**, respectively, shown in Figure 7. Pc 17 was shown to have low cytotoxicity and high uptake in rat osteosarcoma UMR-106 cells [54].

**Figure 8:**
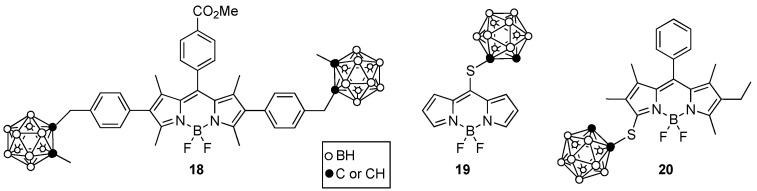
Representative boronated BODIPYs.

Boron dipyrromethenes, known as BODIPYs, have also been investigated as PDT and/or BNCT sensitizers. This type of molecule displays intense absorption and fluorescence emissions similar to those of porphyrin derivatives but has a lower molecular weight and higher solubility. Furthermore, the fluorine atom on the BF_2_ moiety of BODIPYs can be replaced by ^18^F allowing for dual imaging by PET and fluorescence [57]. BODIPYs bearing one or two boron clusters, such as **18**–**20** shown in Figure 8, were synthesized and investigated for their cytotoxicity, cellular uptake, and BBB permeability [58,59]. These studies indicated that these BODIPYs exhibit low toxicity, high uptake into glioma T98G cells, and moderate BBB permeability, particularly for compound **19**, due to its low molecular weight (<400 Da) and enhanced hydrophobicity (log *P* ~1.5). 

## 5. Boron-Containing Liposomes

**Figure 9:**
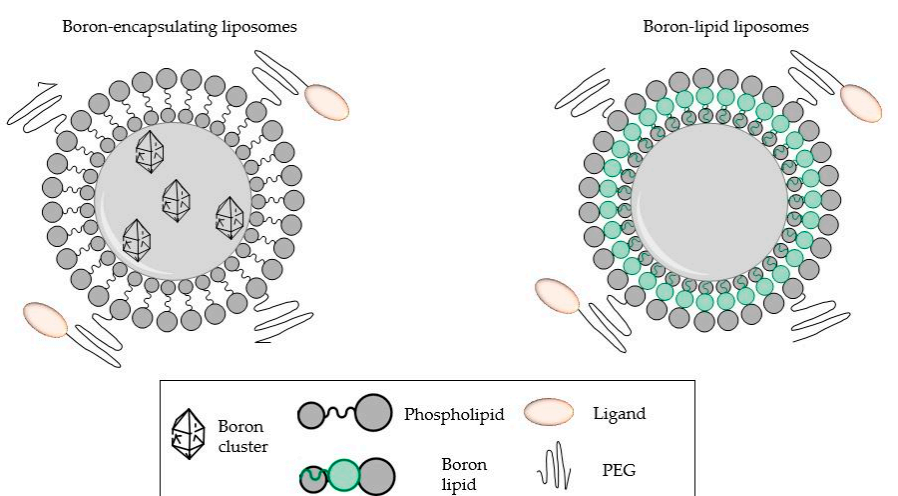
Strategies of boron conjugation to liposomes [63].

Nanoscale carriers, such as liposomes, present a viable strategy for improving the delivery of boron compounds to the targeted tissues. Liposomes offer several advantages as drug delivery systems such as high loading efficiency, proven clinical safety, selectivity towards tumors, easy surface modification, and increased drug stability [60,61,62]. Liposomes are able to encapsulate large amounts of boron drug(s) within their closed lipid bilayers and/or the aqueous core (Figure 9), which allows the controlled release of the ^10^B compounds. Boron-functionalized lipids have also been used as an alternative strategy to incorporate boron [11,63,64]. The enhanced permeability and retention effect (EPR) of liposomes ensures the therapeutic accumulation of boron into the target tumor tissues. Other than ^10^B-containing compounds, liposomes have also been reported as efficient delivery systems for other agents, such as C-X-C chemokine receptor type 4, which targets triple negative breast cancer, and nerve growth factors (into brain tissue), overcoming the blood–brain barrier [65,66].

BSH-encapsulated liposomes were first reported by Yanagie et al. [63]. Liposomes containing ^10^B compound (Cs_2_^− 10^BSH) were conjugated with monoclonal antibodies specific to the carcinoembryonic antigen (CEA). In vitro studies showed the ability of these immunoliposomes to selectively bind to CEA-bearing cells and significantly suppress tumoral growth upon thermal neutron irradiation. Their cytotoxic effect was observed to be dependent upon the density of antibodies conjugated to the liposomes and the concentration of ^10^B delivered. 

Lee et al. encapsulated *nido*-carborane anions into PEGylated liposomes and investigated their activity as BNCT agents [67]. The boronated liposomes were prepared by the thin-film and hydration method, achieving a 47.5 ± 3.1% *nido*-carborane loading. The loading yields were significantly higher than 32.5 ± 1.5% reported by Xu et al. in a similar experiment using hydrophilic boron compounds incorporated into the aqueous core of liposomes [68]. Lee and his co-workers observed a high boron uptake into the tumors relative to normal tissues, which can be attributed to the PEG effect. A PEG hydration layer forms at the surface of liposomes preventing their adsorption into macrophages and serum proteins, ensuring a prolonged residence time for the liposomes in circulation [64]. Liposome uptake in the liver and spleen in the reticuloendothelial system (RES) was low. The viability of the CT26 cancer cells on treatment using 70 μM *nido*-carborane and thermal neutron irradiation (1.94 × 10^4^ /cm^2^·s) was 17.1% compared to the irradiated control cells. In vivo studies on the efficacy of these boronated liposomes on tumor models exhibited slow tumor growth in mice injected with boronated liposomes upon neutron irradiation. The BNCT group treated with 21 mg ^10^B/kg showed 330% tumor growth by volume over 25 days compared to a 3963% increase in the control group and 3952% in the group subjected to neutron irradiation only. These results demonstrate the ability of the synthesized boronated liposomes to significantly suppress tumor growth.

PEGylated liposomes have also been prepared by post insertion where the PEG is added after liposome formation [64]. These were evaluated in comparison to the conventional PEGylated liposomes where PEG lipid is added before the liposome formation. While the post-PEG liposomes used only half of the amount of PEG required for pre-PEG liposomes, their physicochemical properties were found to be similar. Both liposomes had an average size of 100 nm, a polydispersity index of 0.08, an encapsulation efficiency of 68% and equivalent charge densities. In vitro cytotoxicity and cell uptake studies on RAW 264.7 and B16 melanoma cells determined that the PEGylation methods did not affect cell viability. Both methods delivered a therapeutic amount of boron (up to 10^9 10^B atoms/tumor cell) for effective BNCT [69]. The average boron concentration of the liposomes was 73.2–77.6 µg ^10^B/g of tumor tissue, which exceeded the 30 µg ^10^B/g considered sufficient for effective BNCT [8,13].

The BNCT activity of liposomal boron-delivery systems has been investigated on rabbit hepatic cells upon intra-arterial infusion of ^10^BSH [70]. The ^10^BSH-entrapped transferrin-conjugated polyethylene glycol liposomes were prepared with distearoyl-boron lipid (^10^BSH-TF-PEG-DSBL). The TF-conjugated liposomes had previously been observed to be useful for cytoplasmic delivery in vivo [71]. This is due to their ability to easily bind to cancer cells and be internalized by endocytosis, thus facilitating the discharge of liposomes into the tumor tissue. ^10^BSH-PEG-DSBL liposomes and ^10^BSH-PEG liposomes were also prepared for comparison and 15 mg ^10^B/kg of each was administered to tumor-bearing rabbits. The ^10^B accumulation in the VX-2 tumors using DSBL liposomes was two-fold higher than the ^10^B accumulation using ^10^BSH-PEG, after both 24 and 48 h. These liposomes also showed selectivity towards hepatic tumors with the ^10^B concentrations being twice as high as those accumulated in normal hepatic tissues. After 72 h, ^10^BSH-TF-PEG-DSBL liposomes were observed to have 25 μg/g ^10^B concentration in the tumors compared to 10–15 μg/g in the normal hepatic tissues. Reduction of the administered dose of ^10^BSH-TF-PEG-DSBL to 6.4 mg ^10^B/kg maintained the high ^10^B concentration (25 μg/g) 72 h after injection. Suppression of tumor growth was observed after thermal neutron irradiation (2 × 10^12^ n/cm^–2^) 48 h after intra-arterial injection of the ^10^BSH-TF-PEG-DSBL liposome.

Several research groups continue to show liposomal modification with PEG as a promising strategy to overcome barriers in boron delivery for BNCT [72,73,74,75]. However, PEGylation is known to stabilize the liposomes by lowering their interaction with serum proteins and avoiding removal by the reticuloendothelial system. Longevity of liposomes in the bloodstream may result in poor uptake into the targeted cells [76]. Besides transferrin, other tumor targeting moieties such as folate, monoclonal antibodies and epidermal growth factors have also been used in conjunction with liposomes to improve their delivery efficiency [8,64,76]. Folate receptors and epidermal growth factor receptors (EGFR) are overexpressed in tumor cells compared to normal cells. This makes them suitable targets when folate or epidermal growth factor-conjugated liposomes are used for boron delivery [76]. For example, EGFR-targeted PEG liposomes exhibited a high (90 ppm) cellular uptake of ^10^B. The killing effect of the liposome complex on glioma cells was ten-fold higher than that of the neutron-only control cells at an irradiation strength of 3 × 10^12^ n/cm^2^ [77]. Specific binding of these liposomes to HER2 receptors in SK-BR-3 breast cancer cells was also observed. The HER2-targeted ^10^B-containing liposomes achieved 132 ppm intracellular concentrations of ^10^B with 67% retention after 48 h [78].

Cationic liposomes as boron carriers have also been used in BNCT due to their ability to preferentially target the cell nucleus. [79]. Ristori et al. successfully loaded glucosyl-carborane and lactosyl-carborane into charged 1,2-dioleoyl-3-trimethylammonium-propane (DOTAP) and zwitterionic 1,2-dioleoyl-glycero-3-phosphoethanolamine (DOPE) [80]. These cationic liposomes increased the ^10^B concentration in the cells 30 times more than the concentrations observed with ^10^BPA. This is attributed to their favorable electrostatic interactions with the negatively charged outer plasma membranes [81].

Combinational BNCT and chemotherapy were reported by Li et al. based on their boron-encapsulated liposome, boronsome [82]. The carboranyl phosphatidylcholine-based liposome exhibited an intracellular uptake of 182 μg/10^6^ cells, which was 61% higher than BPA. Positron-emission tomography (PET) imaging with ^64^Cu showed long retention in the cells, up to 48 h, with low uptake in normal tissues. The tumor-to-blood ratio was 4 ± 0.3, which is a significant improvement over BPA. In vivo studies using mouse models showed suppressed tumor growth upon treatment with boronsome followed by neutron irradiation, and the therapeutic outcomes were further amplified by encapsulation of chemotherapy drugs.

## 6. Boron-Containing Nanoparticles

Boron-containing nanoparticles (NPs) have lately gained considerable attention as promising delivery systems for tumor-targeted BNCT. NPs have been demonstrated to exhibit selective and high tumor uptake, limiting toxicity to normal tissues, improving cellular stability and delivering high boron content [13,83,84,85]. Elemental boron NPs present a viable tool that can be used to deliver therapeutic amounts of boron into tumors for BNCT due to their high boron content [86]. With the aim of overcoming their lack of tumor-targeting ability and frequent aggregation in aqueous solutions, Zaboronok and co-workers identified hydroxyethyl cellulose (HEC) as a suitable polymeric stabilizer for the NPs [87]. The HEC-stabilized elemental boron NPs were able to deliver high boron concentrations to tumor cells and featured functional groups that could be used to link the NPs to tumor-targeting molecules. Furthermore, the NPs were observed to have high stability in aqueous solutions with no aggregation seen up to 90 days after incubation. This is due to the noncovalent interactions between the HEC polymer and the boron nanoparticles. There was no cell cytotoxicity observed in T98G, U87, and U251 human glioma cells with the minimum therapeutic concentration (20 μg/mL) administered. In vitro studies revealed that the HEC-stabilized elemental boron NPs were superior to BPA as they significantly hindered the colony-forming capacity in all the irradiated cells. Recently, Kaniowski et al. developed functionalized boron-containing NPs capable of localizing in the cell cytoplasm [88]. The complex NPs prepared from 1,2-dicarba-closo-dodecaborane (C_2_B_10_H_12_) conjugated with antisense oligonucleotides showed an ability to penetrate cellular membranes and accumulate in high order structures within the cells. These complexes, upon enrichment with ^10^B atoms, can be used for dual anticancer therapy by downregulating the EGFR oncogene in cancer cells and ^10^B delivery for BNCT.

Gold nanoparticles (AuNPs) are the most used nanoparticles in nanomedicine owing to their unique physicochemical and optical properties, their ease of preparation and surface functionalization. Multi-functional nanosystems have been exploited as valuable tools for targeted drug delivery, controlled release of therapeutic agents, anticancer therapy, cell labeling and imaging, among other applications [89,90,91]. Preliminary studies demonstrated that boron cages can be readily linked to AuNPs, presenting a promising approach capable of delivering sufficient ^10^B atoms for BNCT. Wu and co-workers reported the in vivo distribution of boronated AuNPs in tumors via non-invasive imaging [92]. The AuNPs were PEGylated and modified with carborane cages on the surface and finally linked to an anti-HER2 antibody (61 IgG). The diameter of the active targeting AuNPs was determined by DLS to be 54.48 ± 14.72 nm and the radiolabeling efficiency was 60 ± 5%. Biodistribution studies assessed by ICP-MS indicated antibody-linked AuNPs had the highest T/N ratio (12.02 ± 0.94) which agreed with those derived by MicroSPECT/CT imaging (12.02 ± 0.94). In vitro cellular uptake (19.66 ± 2.71) and internalization assays (15.40 ± 1.72) using N87 human gastric cancer cells indicated higher ratios for the antibody-linked AuNPs compared with non-targeted AuNPs. 

An innovative approach towards developing tumor-selective gold nanoclusters containing *nido*-carborane amino derivatives (GNCs-CB) was reported by Wang et al. [93]. The theranostic ability of this delivery nanosystem was demonstrated by precise tumor imaging and prolonged tumor accumulation. This allowed for accurate tumor-targeted delivery of the boron-rich nanoclusters for BNCT and enabled the monitoring of the process by fluorescence imaging. Recently, albumin-AuNP theranostic agents were prepared by conjugation of undecahydro-closo-dodecaborate (B_12_H_12_) or anticancer nucleotide trifluorothymidine (TFT) to bimodal human serum albumin, linking the derivatives with AuNPs [94]. Boron-enriched AuNPs bearing radiolabeling isotopes (^124^I or ^64^Cu) at the core or shell have also been investigated for their tumor accumulation and biodistribution properties [95,96,97]. Labeling NPs enables the simultaneous delivery of boron atoms for therapeutic purposes as well as determining the ^10^B concentration in specific tissues via non-invasive methods [13,98].

Magnetic drug-targeting methods are based on attaching therapeutic agents onto delivery systems that are magnetically responsive [99,100]. This enables the precise targeting of tissues using a magnetic field and the process is monitored in real-time by imaging. For example, iron-boron (Fe-B) NPs have been used for their multiple functions in BNCT guided by magnetic resonance imaging (MRI) [101]. The Fe-B NPs possess high neutron capture therapy ability from the boron, coupled with the magnetic properties of iron. This enables MRI-guided localization of ^10^B atoms in the target tissues, assisted by magnetic hyperthermia and magnetophoretic accumulation [102]. Recently, magnetic NPs have been based on iron oxides attached to a carborane cage [102,103,104,105]. Korolkov et al. successfully attached isopropyl-*o*-carborane on Fe_3_O_4_ NPs pre-modified on the surface with tetraethoxysilane (TEOS) and (3-glycidylpropyl) trimethoxysilane (GPTMS) [105]. These NPs were characterized by various spectroscopic methodologies for their morphology, structure, and chemical composition. Cytotoxicity assays were performed in vitro using various human cell lines for prostate, pancreatic, colon and cervical cancer. The results indicated non-toxicity of the NPs prepared in concentrations lower than 200 µg/mL. These studies showed desirable biocompatibility of the NPs with the tissues which facilitates their degradation in the lysosomes and clearance from the cells.

Mesoporous materials such as silica NPs are favored as boron-delivery vehicles due to their easily tunable size, large mesopore volume for high boron-loading capability, easy surface functionalization and biocompatibility for both in vitro and in vivo studies [106,107]. Recently, Wang et al. designed carborane-enriched dendritic mesoporous silica nanospheres (DMSNs) that were able to selectively target the overexpressed integrin receptors in pancreatic tumor sites [106]. The mesopores were loaded with doxorubicin (DOX), and both a fluorescence tracer and an anticancer drug for synergistic treatment of cancer by BNCT and chemotherapy. Wang and his colleagues achieved a high boron-loading capacity of 141.5 mg/g enabling the delivery of sufficient amounts of ^10^B atoms (24.4 μg ^10^B/g) to the tumor cells. Theranostic ^10^B-enriched boron NPs coated with a thin layer of silica to form ^10^B-SiO_2_ NPs were also investigated for BNCT in glioblastoma multiforme (GBM) cells [108]. Silica coating improved the biocompatibility of the NPs and enabled surface modification with fluorescein isothiocyanate (FITC)-labeled RGD-K peptide. The NPs were able to selectively target GBM tumor cells, delivering a large amount (50.5 μg ^10^B /g) of ^10^B to the cells. MRI-guided in vivo studies significantly suppressed murine brain tumors and increased the half-life of mice from 22 days to 39 days.

Since the discovery of carbon nanotubes (CNTs), significant attention has been drawn towards them owing to their unique properties, such as excellent conductivity, thermal stability, nanoscaled size and their potential applications in biomedical nanotechnology [109,110]. Both single-walled CNTs and multi-walled CNTs have been studied for applications as drug delivery systems. Due to their size (100–300 nm), functionalized CNTs avoid uptake by the RES prolonging their circulation in the blood stream as well as improving their bioavailability. Gao and co-workers designed *nido*-carborane units supported on water-soluble single-walled CNTs [111]. These nanocomposites were tumor specific, delivering 21.5 µg ^10^B/g into the tumor within 48 h of administration. However, water solubility and biocompatibility are the main challenges faced by CNT-based delivery systems. Boron nitride nanotubes (BNNTs), which are structural analogs of CNTs, have been considered as a better alternative candidate for BNCT applications. BNNTs feature high boron density (approximately 50%) equivalent to hundreds or thousands for each nanotube [111,112]. Further, boron-enriched BNNTs have superior thermal and chemical stability compared to CNTs, in addition to their ability to resist oxidation and superior biocompatibility [13]. In a bid to enhance the selective targeting of GBM, Ciofani et al. reported folate-conjugated BNNTs coated with biocompatible poly-l-lysine [113]. The BNNTs were further functionalized with a fluorescent probe to enable tracking. In vitro studies showed the nanotubes selectively localized in the GBM cells. Covalent grafting of transferrin onto BNNTs has also been successfully achieved demonstrating enhanced cellular uptake in human endothelial cells [114]. This has significant potential in accessing the brain through the BBB as transferrin receptors are highly expressed in brain cells. Another study by Nakamura and co-workers reported methoxy-poly(ethyleneglycol)-1,2-distearoyl-sn-glycero-3-phosphoethanolamine functionalized BNNT (BNNT-DSPE-PEG2000) that accumulated in B16 melanoma cells three times higher than BSH, delivering a high BNCT antitumor effect [115]. 

Boron phosphates (BPO_4_) are inert materials that can be prepared as NPs with a ^10^B concentration at approximately 10%. Preliminary studies investigated the compatibility of folic functionalized BPO_4_ NPs against different types of cells [116]. Cytotoxicity studies on rat neoplastic cell lines were also performed with the folate-BPO_4_ NPs and the results were comparable to BPA indicating the potential of functionalized BPO_4_NPs as a boron-delivery agent. Improving on these findings, ^10^B-enriched BPO_4_NPs (98.5% ^10^B loading) were synthesized and modified on the surface with anti-EGFR antibodies equipping them with tumor-targeting ability [117]. A high accumulation of ^10^B atoms (63 μg ^10^B/g tumor tissue) was observed in the head and neck cancer tumor sites and the T/B ratio was 4.27. The anti-EGF-^10^BPO_4_ NPs induced 72% cellular death compared to only 30% observed using BPA.

For effective BNCT, most of the current delivery strategies rely on ^10^B-enriched boron precursors to achieve therapeutic boron levels in the tumor cells. To avoid the high costs associated with the ^10^B enrichment process, Chiang et al. prepared non-^10^B-enriched polymer-coated boron carbon oxynitride (BCNO) NPs to conduct a BNCT theranostic study [118]. BNCO is a non-toxic earth phosphor that requires no additional labeling modifications for its applications, due to its photoluminescence property. Chiang and his co-workers were able to modulate the optical, cellular uptake and cytotoxicity properties to achieve sufficient tumoricidal effect. Recently, studies have been carried out to investigate the co-assembly of BNCO NPs within acid-labile polyethylene glycol-graft-polyethyleneimine (PEG-g-PEI) DHGC [119]. Change in the *zeta* potential and hydrodynamic size of the BNCO/DHGC nanoparticle complex was also investigated at different mixing ratios. Stable aqueous dispersion was achieved for all the mixing ratios. To modulate the surface charge and size of the nanoassemblies, the mixing ratio, ionic strength, molecular weight and pH of the solution can be adjusted. Electrostatic colloidal co-assembly therefore proved a potential synthetic tool useful in developing hybrid nanosystems for effective BNCT applications.

Recently, additional multi-functional boron-delivery systems for BNCT have been proposed. These materials are being designed to possess superior properties in biocompatibility, immune evasion, bioimaging and active cell targeting. Bio-mimicking principles with cell membranes derived from optimized cells have been used to prepare exosome-coated boron containing carbon dots (BCDs) [120,121]. Carbon dots have good photoluminescence properties, attracting potential applications in tumor theranostics. Li and his co-workers reported enhanced T/N ratios and a curative BNCT effect on brain glioma cells using exosome encapsulated BCDs [121]. Fluorescence-guided imaging with these nanosystems achieved an excellent match between boron and neutron exposure. Wang et al. designed metal-organic frameworks (MOFs) from zirconium *meso*-tetra(4-carboxyphenyl)porphyrin (Zr-TCPP) [122]. TCPP, a porphyrin derivative, enabled selective cellular uptake in the tumor with high accumulation efficiency. Optimized Zr-TCPP MOFs with suitable size were selected to achieve a maximum loading of boric acids and studied for BNCT applications. The excellent fluorescence properties of TCPPs enabled visualization and accurate boron quantification which improved the curative effect of BNCT on glioma cells studied by Wang and his group.

## 7. Conclusions

Tumor-targeted boron-delivery agents that are radio-labeled or linked to a fluorophore, and able to deliver a therapeutic amount of ^10^B to tumors with high T/N and T/B, are promising multi-functional agents for BNCT. On the other hand, non-targeted boron compounds, including the two clinically approved drugs for BNCT, show enhanced tumor accumulation mainly due to the enhanced permeability and retention (EPR) effect, but this leads to suboptimal T/N and T/B, which can cause adverse treatment side effects. Cell surface receptors overexpressed in cancer cells are promising targets for enhanced selective tumor therapy. Monoclonal antibodies (mAbs) and peptides are among the most effective tumor-homing agents, and their conjugation with boron-delivery agents has produced promising agents. While mAbs efficiently target cell surface receptors, such as EGFR, their tumor cell internalization is limited and often does not lead to significant boron localization in key organelles, including the cell mitochondria and nuclei. Smaller biomolecules, such as peptides, are an attractive alternative. Peptides are easy to synthesize and functionalize and can be rapidly cleared from blood and tissues by proteolytic enzymes. To ensure persistence in the tumor during treatment, several strategies can be adopted to increase their stability, including the use of D-amino acids. Both cell-targeting and cell-penetrating peptides have been functionalized with boron clusters leading to promising conjugates with enhanced biological properties relative to the clinically approved BNCT agents. Of these, the cell-targeting peptides tend to be more effective in the selective delivery of boron to tumors.

Another promising strategy is the use of a boronated agent that allows the dual therapy of cancer, for example, chemotherapy-BNCT and PDT-BNCT. In the latter, the use of porphyrin derivatives is very promising, as they are well-known photosensitizers and have been widely used in clinical photodynamic therapy. In addition, porphyrin derivatives can easily be conjugated to peptides, radio-labeled, and/or complexed with ^64^Cu(II) and other metal ions, allowing for PET imaging and quantification of boron localized in tissue.

Finally, nanoparticles and liposomes are highly promising multi-functional agents with the ability to carry large boron loads and other molecules. Considerable success has been demonstrated using these strategies, resulting in improved cellular uptake of ^10^B atoms, high tumor–boron accumulation, and precise targeting of overexpressed receptors in tumor cells. The theranostic capabilities of the nanomaterials have been demonstrated, enabling the dual function of delivering a therapeutic dose of ^10^B atoms for effective BNCT as well as monitoring of the process. The ease of preparation and functionalization of liposomes and nanoparticles, along with their biocompatibility properties present a promising avenue that may overcome the current challenges of BPA and BSH in clinical trials.
